# Drinking Tremor

**DOI:** 10.1002/mdc3.70398

**Published:** 2025-10-15

**Authors:** Victor Rebelo Procaci, Lyamara Apostólico de Azevedo, Orlando Graziani Povoas Barsottini, Henrique Ballalai Ferraz, José Luiz Pedroso

**Affiliations:** ^1^ Department of Neurology Universidade Federal de São Paulo São Paulo Brazil

**Keywords:** drinking tremor, orolingual tremor, task‐specific tremor, tremor

## Question

**FIG. 1:**
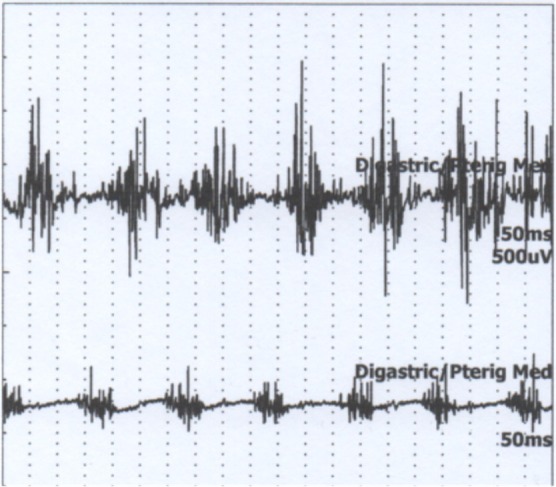
Electromyography (EMG) of the anterior digastric and medial pterygoid muscles during drinking from a glass, showing rhythmic bursts at ~7 Hz. Full report available in Data S1, Supplementary appendix.

An 80‐year‐old woman presented with a 5‐year history of jaw tremor occurring exclusively when drinking liquids from a glass. The tremor improved significantly when using a straw (Video 1). No tremor was observed at rest or during speech. There were no signs of parkinsonism, dystonia, or tremor in the arms, and the remainder of the neurological examination was unremarkable. She reported no comorbidities and was not taking antidopaminergic medications. The patient also denied playing wind instruments or having undergone invasive dental procedures. Electromyography (EMG) demonstrated rhythmic bursts of activity consistent with a task‐specific tremor at approximately 7 Hz, with burst durations ranging from 50 to 100 ms, initiated when drinking from a glass (Figure 1; Data S1). Activation was more prominent and intense in the anterior digastric muscle compared with the masseter and medial pterygoid muscles.

**Video 1:** (Part 1) No tremor is observed at rest. Tremor appears when the patient drinks from a glass and is markedly reduced when drinking through a straw. (Part 2) During the act of drinking from a glass, electromyography (EMG) demonstrated rhythmic bursts of activity consistent with a task‐specific tremor at approximately 7 Hz, with burst durations ranging from 50 to 100 ms. Activation was more prominent in the anterior digastric muscle. No co‐contraction of agonist and antagonist muscles was observed, excluding a dystonic pattern. When drinking with a straw, a slight onset of involuntary movement was initially noted, but once the straw was fixed between the lips, the tremor disappeared.

## What Is the Most Likely Diagnosis?

(1) Jaw tremor in Parkinson's disease
(2) Essential tremor
(3) Task‐specific orolingual tremor
(4) Dystonic tremor

## Answer

Task‐specific orolingual tremor is a rare tremor syndrome characterized by involuntary, rhythmic oscillations of the orolingual muscles that occur exclusively during a specific task. Its etiology is often idiopathic, and the condition can be both disabling and socially stigmatizing. Therapeutic options include botulinum toxin injections, propranolol, and primidone, although their efficacy is limited.^1^ Clinical history should include questions about playing wind instruments and prior dental procedures, because these have been described as potential associated factors.^2^ It is also important to assess for alleviating maneuvers, such as in our case, where the patient experienced significant improvement when drinking through a straw. Our patient did not improve with propranolol, primidone, or botulinum toxin injections, and ultimately opted to use a straw to alleviate the drinking tremor.

## Author Roles

(1) Research project: A. Conception, B. Organization, C. Execution; (2) Statistical Analysis: A. Design, B. Execution, C. Review and Critique; (3) Manuscript: A. Writing of the First Draft, B. Review and Critique.

V.R.P.: 1A, 1B, 1C, 3A

L.A.d.A.: 1B, 3B

O.G.P.B.: 3B

H.B.F.: 3B

J.L.P.: 1A, 1B, 3B

## Disclosures

**Ethical Compliance Statement**: This study was approved by our local ethics institution. Patient consent form was obtained. We confirm that we have read the Journal's position on issues involved in ethical publication and affirm that this work is consistent with those guidelines.

**Funding Sources and Conflict of Interest**: No specific funding was received for this work. The authors declare that there are no conflicts of interest relevant to this work.

**Financial Disclosures for the Previous 12 Months**: Dr. Victor Rebelo Procaci has received honoraria from Biogen for delivering an educational lecture within the past 12 months. The authors report no other relevant financial disclosures or conflicts of interest.

**Potential Conflict of Interest or Financial Disclosure**: The Article Processing Charge for the publication of this research was funded by the Coordenação de Aperfeiçoamento de Pessoal de Nível Superior ‐ Brasil (CAPES). The authors declare no other conflicts of interest related to this article.

## Supporting information


**Data S1.** Supplementary appendix. Full EMG report.

## Data Availability

Data sharing not applicable to this article as no datasets were generated or analysed during the current study.
