# Supplementary material for: Drinking Tremor
Source: Mov Disord Clin Pract. 2025 Oct 15;13(3):830–1. doi: 10.1002/mdc3.70398 (PMC13042513; doi:10.1002/mdc3.70398)
Supplement: Supplementary file 2 — Data S1. Supplementary appendix. Full EMG report. [file MDC3-13-830-s001.docx]

**Supplementary appendix**

**Electromyography (EMG) Report**

A concentric disposable needle electrode was used to evaluate the following muscles bilaterally: anterior digastric, mylohyoid, masseter, medial and lateral pterygoids, temporalis, mentalis, orbicularis oris, and genioglossus.

At rest, no signs of acute denervation (fibrillations or positive sharp waves) were observed in the examined muscles. During voluntary contraction, interference patterns, firing frequency, and motor unit recruitment were normal across all muscles tested. No motor unit action potentials with myopathic or neurogenic characteristics were detected.

The involuntary movement was assessed during placement of a glass to the mouth and initiation of drinking. Alternating contraction of agonist and antagonist muscles involved in mouth opening and closing was observed, consistent with a regular task-specific tremor at 7 Hz, with burst durations ranging from 50 to 100 ms. Activation was more evident and intense in the anterior digastric muscle compared with the masseter and medial pterygoid muscles. With the use of a straw, a slight onset of involuntary movement was initially observed, but once the straw was fixed between the lips, the movement disappeared. No co-contraction of agonist and antagonist muscles (as seen in dystonia) was observed.

Conclusions:

Electromyographic examination demonstrated a task-specific action tremor with a frequency of approximately 7 Hz. No co-contraction of agonist and antagonist muscles (dystonia) was identified.

**Additional references suggested by reviewers:**

Ghadery CM, Kalia LV, Connolly BS. Movement disorders of the mouth: a review of the common phenomenologies. J Neurol. 2022 Nov;269(11):5812-5830. doi: 10.1007/s00415-022-11299-1. Epub 2022 Jul 29. PMID: 35904592.
